# Implementation of single-item alcohol screening and brief intervention in a primary care clinic in western Colorado

**DOI:** 10.1186/1940-0640-10-S2-P8

**Published:** 2015-09-24

**Authors:** Kelly Marzano, Carolyn Swenson

**Affiliations:** 1 OMNI Institute, Denver, CO 80209, USA; 2 Peer Assistance Services, Denver, CO, 80231, USA

## Background

The U.S. Preventative Task Force recommends screening and brief intervention (SBI) as an effective strategy to address risky alcohol use[1]. Yet, only about 25% of binge drinkers have talked to a health care professional about alcohol use[2]. Perceived lack of time, difficulty implementing SBI protocols into clinic flow, and staff buy-in have been identified as barriers to successful implementation. We conducted a pilot study in a 3-physician primary care practice to evaluate successes and challenges in implementing a simple approach to alcohol SBI in primary care.

## Materials and methods

A primary care clinic in Colorado implemented a single-item alcohol screening question. Screening was only implemented for patients with certain appointment types (e.g., new patients, physical exams, visits for depression and anxiety). Patients who screened positive received a brief intervention provided by the physician. SBI data were documented in the electronic medical record. Patients scored positive when they indicated binge drinking in the past three months (more than 3/occasion for women; more than 4/occasion for men). Physicians and staff participated in a site visit, staff focus group, and provider interviews.

## Results

Of the 1190 patients with designated appointments types, only 53.8% (n=640) had a screening result recorded. Of appointments for which a response was recorded, 19.4% (n=124) scored positive, and 47.6% (n=59) of those with a positive screen received a brief intervention. In addition, 1.9% (n=10) of patients who scored negative were provided a brief intervention based on physician assessment of alcohol use. Staff identified that the screening question was easy to administer, and that it led to increased recognition among patients about level of alcohol consumption, and to improved care. Nonetheless, challenges in consistently adminstering the question and following up on positive screens, and in identifying appropriate resources for referrals, were noted.

## Conclusions

Results suggest that there are benefits in implementing a single-item screening question for alcohol use. Nonetheless, challenges exist in consistently administering even a very simple SBI process in practice.
